# Working memory capacity as a moderator of load-related frontal midline theta variability in Sternberg task

**DOI:** 10.3389/fnhum.2014.00399

**Published:** 2014-06-06

**Authors:** Marta Z. Zakrzewska, Aneta Brzezicka

**Affiliations:** Department of Psychology, Interdisciplinary Center for Applied Cognitive Studies, University of Social Sciences and HumanitiesWarsaw, Poland

**Keywords:** frontal midline theta, EEG, working memory capacity, neural efficiency, individual differences

## Abstract

The aim of this study was to investigate the relationship between working memory capacity (WMC) and frontal theta response to memory load in Sternberg task. We show that oscillatory activity in the theta band (4–6 Hz) related to Sternberg task performance may differentiate people characterized by high and low WMC. Specifically, there is a linear increase of frontal midline (FM) theta power with load, however, only in the high WMC group. Furthermore, a positive linear relationship was found between WMC (operation span task score) and average FM theta power increase from lower to higher loads which was not present at other scalp locations. The distinct patterns of high and low WMC individual’s FM theta response to memory load seem to support the assumption that theta activity during maintenance reflects not only the amount of information stored, but also the effort it takes to remember them and the efficiency of involved neural processes. This contributes to perceiving FM theta as an individual trait which can reflect individual working memory mechanism efficiency.

## INTRODUCTION

Oscillatory activity in the theta band (4–8 Hz) is traditionally associated with various memory and learning processes. Among these processes are: long term potentiation (LTP; Holscher et al., 1997), encoding of new information (Klimesch, 1999; Hasselmo, 2006) and successful maintenance of information in WM (Itthipuripat et al., 2013). An increase of frontal midline (FM) theta synchronization can be also observed in subjects performing tasks requiring cognitive and mental effort (Gevins et al., 1997). For example, theta power increases with memory load in Sternberg task (Gevins et al., 1997; Onton et al., 2005) and n-back task (Klimesch et al., 2005). This effect is present regardless of task modality. Interestingly, individuals differ in the degree to which they show this task load-related theta increase. FM theta variability has been linked to personality traits such as anxiety, extroversion, and neuroticism (Inanaga, 1998). It has also been shown that people differ in the visibility of FM theta (Mitchell et al., 2008) which suggests that FM theta could be a viable marker of individual differences in these aspects of behavior, which are related to functioning of fronto-midline parts of the brain. As this part of the brain is crucial for behavioral control, broadly defined working memory or executive functions among others, it is quite surprising that so far FM theta has not been directly linked to strictly cognitive individual characteristics except for one study (Gevins and Smith, 2000). The study described in this article is the first attempt to recognize the role of individual differences in working memory capacity (WMC) in FM theta variability. WMC is a set of basic cognitive skills crucial for execution of many higher order cognitive functions, such as planning (Barrett et al., 2004), learning (Cantor and Engle, 1993), reading and listening comprehension (Daneman and Carpenter, 1980), problem solving (Adams and Hitch, 1997) or constructing complex and integrated representation of newly acquired material (Rosen and Engle, 1997; Barrett et al., 2004). What is an important, higher WMC results not only in more items being maintained as active part of a memory buffer, but also determines greater ability to control attention and more efficient distraction filtering (Engle, 2002). WMC is also strongly associated with fluid intelligence (Kyllonen and Christal, 1990; Barrett et al., 2004), some researchers claim that this two concepts may in fact reflect the same underlying process (Kane and Engle, 2002; Burgess et al., 2011).

Because of its significance on memory engaging processes, WMC is likely to influence FM theta variability. We postulate that higher WMC might be associated with more efficient information processing (and underlying neural activity) and therefore with distinct frontal theta activity. For these reasons our study aimed at comparing psychophysiological indices of memory load-related response among people with high and low WMC.

## MATERIALS AND METHODS

### PARTICIPANTS

Eighty-one young healthy adults (47 women, 34 men; age *M* = 23, SD = 3.69) were primarily included in this study. All the procedures used in this study had an approval from the Ethical Board of the University of Social Sciences and Humanities. Our inclusion criteria were: completion of operation span task (OSPAN), no history of a neurological disorder and no use of substances such as alcohol prior to research session. From this set, six participants were excluded from the analysis: four due to low math accuracy in OSPAN task (<80%), and two on account of bad electroencephalography (EEG) signal. After splitting the entire group into low and high WMC subgroups (splitting point: median OSPAN score) we excluded another six participants whose scores were equal to or near the median value in order to have low and high WMC groups differentiated. Therefore the number of subjects in the described study narrowed down to 69 (40 women, 29 men, age *M* = 23, SD = 3.46).

### APPARATUS AND MATERIAL

#### Sternberg task

Subjects performed a classic Sternberg paradigm (Sternberg, 1966) during which we recorded EEG signal. Each trail of the task consisted of a set of 2 to 5 white digits presented in a sequence (1200 ms each). 2500 ms after the last digit (maintenance period) a yellow probe digit appeared and subjects had to indicate whether it had been present in the previously displayed sequence or not by pressing an adequate button. Participants were instructed to refrain from any kind of movements and eye blinking except from the time indicated within the task. A visual feedback (green “O” for correct answers, red “X” for incorrect answers) informed subjects whether they answered correctly or not. Task sessions were divided into equally distributed positive (“in,” probe present in the memory sequence) and negative (“out,” probe not present in the memory sequence) trials (120 experimental trials in total, preceded by 15 trials in the training session).

#### Operation span task – WMC assessment

Participants performed an operation span (OSPAN, Unsworth et al., 2005) – without EEG recording – where they were asked to solve simple math equations while simultaneously trying to remember series of letters. Two short training sessions, one for solving math equations, another for remembering letters preceded the actual task trials. In each trial subjects had to solve a math operation during a limited amount of time. Once they solved the operation they were presented with a to-be-remembered letter for 1 s. As soon as the letter disappeared from the screen another math operation was presented. After a set of two to seven operation-letter pairs subjects had to recall letters from the current set by clicking on the letter board displayed on the monitor. Letters had to recalled in the order of appearance therefore a correct answer required indicating correctly both the letters and their order. The score was the sum of items in all correctly recalled sequences. Trials with mistakes (even one) were not included in the total score. Subjects were informed about the importance of solving the equations correctly. We used this behavioral measure to estimate subjects’ individual WMC. Both Sternberg and OSPAN task were administered using Inquisit software (millisecond).

### ELECTROENCEPHALOGRAPHIC RECORDINGS

Electroencephalography registration was done using 64-Channel EGI HydroCel^TM^ Geodesic Sensor Net, NetStation software and an EGI Electrical Geodesic EEG System 300 amplifier. Input impedance was kept below under 40 kΩ before the recordings. We used Cz electrode as reference.

### DATA ANALYSES

**FIGURE 1:**
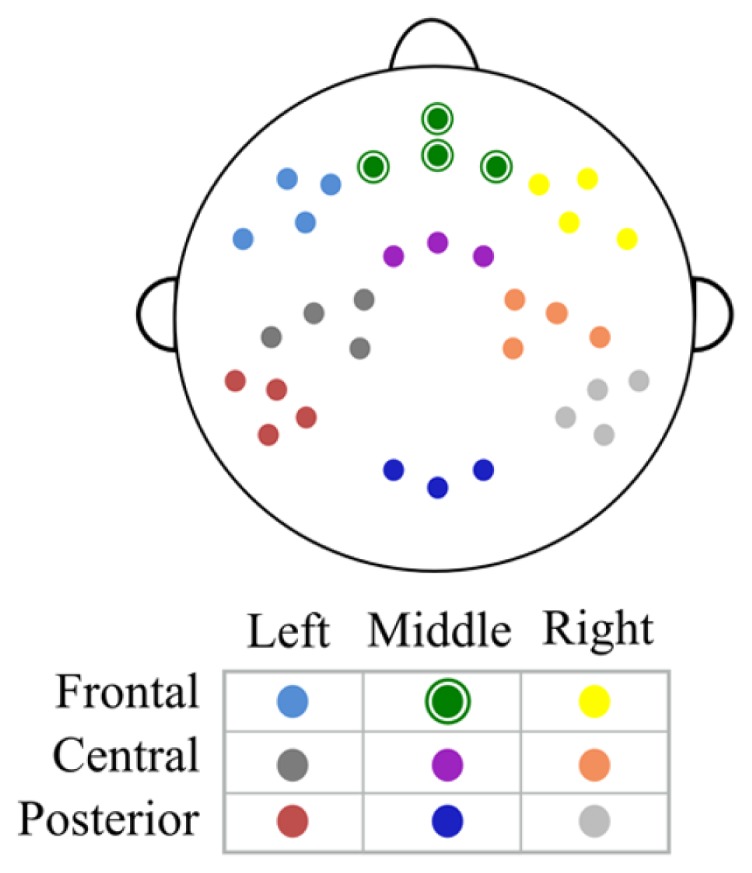
**Scalp topography of middle frontal and remaining electrode clusters.** All clusters taken into analysis. Middle frontal cluster is marked with green, circled dots.

Data analysis was performed off-line using Netstation software, EEGLAB toolbox (Delorme and Makeig, 2004) and custom MATLAB (MathWorks) scripts. The continuous EEG was re-referenced to the average of both mastoids, filtered using a 0.3 Hz high – pass filter in NetStation and exported to EEGLAB. Data was then baseline-corrected, divided into 2500 ms epochs from the maintenance period of Sternberg task and visually inspected for artifacts. Incorrect trials – these in which participants gave incorrect answers – were very rare and excluded from analysis. The average number of incorrect trials varied from 3.6 in high WMC group to 7.2 in low WMC group – too few to use them for analysis. Following removal of epochs with major artifacts (with an average of 84% of epochs remaining) an independent component analysis (ICA) was used in order to identify eye-movement and eye blink related components, which were removed from the signal. Then we estimated the power spectrum (using Welch’s power spectral density estimate) for each electrode and subject separately for every memory load (2, 3, 4, or 5). This power spectrum was averaged in the frequency (4–6 Hz theta band) and area (frontal electrodes: E3, E6, E8, E9) of interest. Power spectrum was computed from the last 1.5 s (1000–2500 ms) interval of the maintenance period in order to avoid the activity evoked by the appearance of the last digit. Apart from averaging the absolute power spectrum, we also computed theta power (same frequency range and area of interest) relative to mean 1–45 Hz power from all loads in order to examine changes in relative theta power. Additionally, we computed absolute and relative theta power for eight other electrode clusters: two frontal clusters (left: E18, E19, E13, E14; and right: E60, E59, E57, E56), three central clusters (left: E15, E16, E20, E22; middle: E7, E4, E54; and right: E51, E50, E41, E49) and three posterior clusters (left: E25, E26, E28, E27; middle: E33, E36, E38; right: E42, E45, E46, E48). Scalp topography of all clusters is depicted in **Figure 1**.

## RESULTS

### BEHAVIORAL RESULTS

#### Sternberg task

Overall, participants performed very well on the Sternberg task (average performance accuracy: 95.7%, SD = 5.33). Mean Sternberg accuracy in the high WMC group was 97% (+/-4) and 94% (+/-6) in the low WMC group. The averaged median reaction times for each group and each load condition are presented in **Table 1**.

**Table 1:** Averaged median reaction times (ms) in each memory load of the Sternberg task for low and high WMC groups.

	WMC	
	Low	High
Load 2	775.93 (221)	725.33 (167)
Load 3	821.06 (192)	778.49 (166)
Load 4	877.16 (202)	819.17 (187)
Load 5	894.68 (220)	838.23 (178)

#### Operation span task

Average OSPAN score was 36,60 (SD = 16,42) with a median at 36 (min = 6, max = 75). We used median as a splitting point between low and high WMC groups ending up with 34 (18 women, 16 men; age *M* = 23, SD = 3.32) subjects in low WMC group and 35 (22 women, 13 men; age *M* = 23, SD = 3.65) in high WMC group. Subjects with scores equal or close to median (35–37) were excluded from the analysis.

#### Sternberg task and working memory capacity

In order to investigate whether reaction times in Sternberg task vary between the two WMC groups we performed a 4 (memory load: 2 vs. 3 vs. 4 vs. 5) × 2 (WMC: high vs. low) repeated – measures ANOVA on individuals’ median reaction time for each memory load. The main effect of memory load was significant [*F*(3,201) = 59.64, *p* < 0,000001, η^2^ = 0.47] unlike the interaction effect [*F*(3,201) = 0.46, *p* = 0.82, η^2^ = 0.004] or a main effect of WMC. Therefore, WMC did not determine participants’ speed of answering.

### ELECTROENCEPHALOGRAPHIC DATA ANALYSIS

In order to examine whether WMC affects load-related changes in theta power we performed a 4 (memory load: 2 vs. 3 vs. 4 vs. 5) × 2 (WMC: high vs. low) repeated – measures ANOVA on theta power averaged over frontal electrodes (E3, E6, E8, E9; **Figure 1**, green cluster) in each load in the theta (4–6 Hz) band. Only significant effects involving memory load are reported. Greenhouse–Geisser correction for violation of sphericity assumption was used when applicable (Greenhouse and Geisser, 1959). We did this for both absolute and relative theta power.

#### Memory load, WMC, and oscillatory activity

**FIGURE 2:**
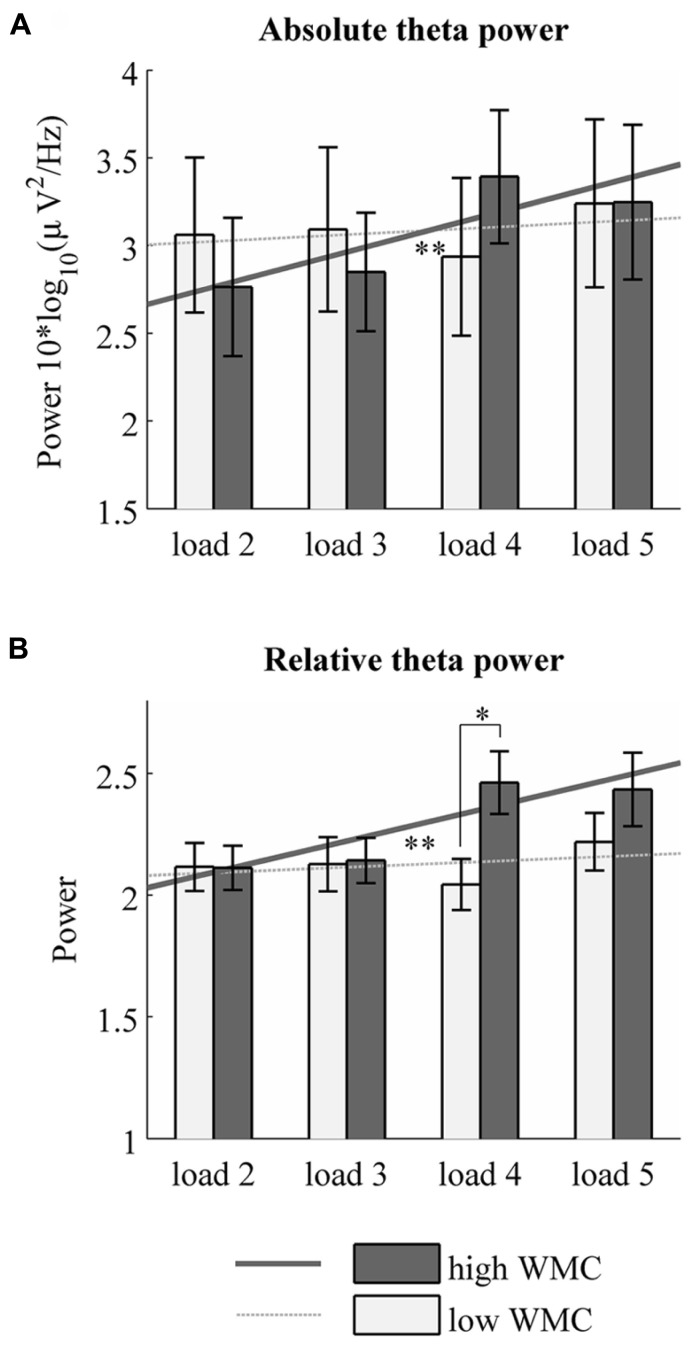
**Absolute and relative theta power in each memory load for low and high WMC groups.** Mean 4–6 Hz absolute **(A)** and relative **(B)** theta power over frontal electrodes in high and low WMC groups. ^*^p < 0.05, ^**^p < 0.01.

The main effect of memory load was significant for both absolute [*F*(3,201) = 3.70, *p* = 0.013, η^2^ = 0.052] and relative [*F*(3,201) = 3.80, *p* = 0.018, η^2^ = 0.054] theta power. Absolute and relative theta power both increased linearly with memory load [absolute: *F*(1,67) = 8,68, *p* = 0.004, η^2^ = 0.115, relative: *F*(1,67) = 7,94, *p* = 0.006, η^2^ = 0.106]. These results are in line with previous findings showing that the systematic increase of theta power with memory load is characteristic for midline frontal regions (Jensen and Tesche, 2002; Onton et al., 2005). Furthermore, we observed a significant interaction between memory load and WMC for absolute [*F*(3,201) = 4,14, *p* = 0.007, η^2^ = 0.058] as well as relative [*F*(3,201) = 3,71, *p* = 0.02, η^2^ = 0.052] theta power. Additional analysis revealed that the abovementioned results differ among people with high and low WMC (**Figure 2**). A repeated measures ANOVA performed separately for high and low WMC group showed that the linear increase of absolute theta power with memory load is specific only for the high WMC group [linear trend: *F*(1,34) = 10.9, *p* = 0.002, η^2^ = 0.244]. We did not observe this effect in the low WMC group [linear trend: *F*(1,33) = 0.51, *p* = 0.47, η^2^ = 0.015]. Same patter was visible for relative theta power [linear trend in high WMC group: *F*(1,34) = 7.784, *p* = 0.009, η^2^ = 0.186, linear trend in low WMC group: linear trend: *F*(1,33) = 0.719, *p* = 0.403, η^2^ = 0.021].

Specifically, in the high WMC group, absolute theta power rises from memory load 2 to load 4 and load 5. No such pattern was found in low WMC group. These results suggest that the dynamics of neural correlate of memory load – theta power – differs depending on one’s WMC. In the high WMC group, theta power in the lowest loads (load 2 and 3) did not differ from each other but was significantly lower than theta power in high loads (load 4 and 5) for both relative and absolute power estimates. High loads did not differ from each other. *p*- values of the aforementioned differences are depicted in **Table 2**. Although there were no differences in absolute theta power between low and high WMC groups, the latter had higher relative theta power in high load condition (load 4) than the former (*p* = 0.014, see **Figure 2B**).

**Table 2:** Load-related theta power increases in high WMC group.

		**Absolute theta power**			
	Load	2	3	4	5
	2		ns.	***	**
**Relative theta power**	3	ns.		**	*
	4	***	**		ns.
	5	**	*	ns.	

Asterisks indicate increase of power from lower to higher load. *p < 0.05, **p < 0.01, ***p < 0.001, ns.-no differences between loads.

**FIGURE 3:**
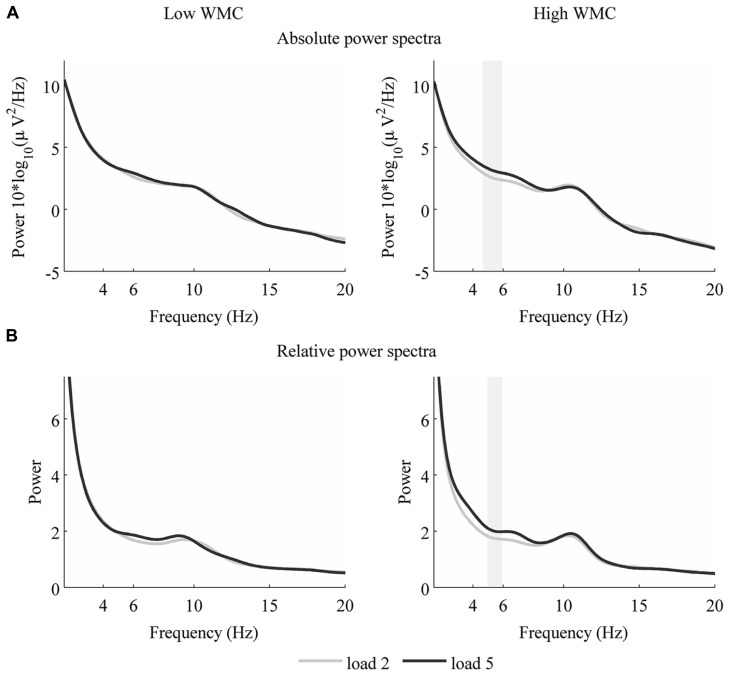
**Power spectrum for loads two and five – for low WMC (left) and high WMC (right) group absolute (A) and relative **(B)** theta power from loads two and five.** Shaded area indicates significant differences in power between the two loads (*p* < 0.05).

To examine this further, we performed a non-parametric statistical test with cluster correction for multiple comparisons to compare spectra from low and high loads (Maris and Oostenveld, 2007). Specifically, we took the entire power spectrum up to 45 Hz from low load maintenance (load 2) and compared it with high load maintenance (load 5). These tests revealed that in the high WMC group EEG activity over frontal sites differs only in theta frequency (absolute theta power: 4.47–6.23 Hz, *p* = 0.04, **Figure 3A**; relative theta power, 4.95–5.92 Hz, *p* = 0.041, **Figure 3B**). Once again, low WMC group’s signal didnot change between loads (**Figure 2**).

#### Changes in theta power and operation span

**FIGURE 4:**
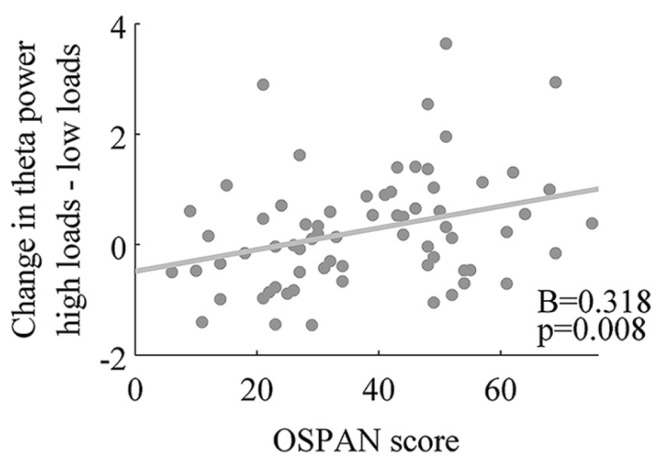
**Positive association of working memory capacity and theta power change from low loads to high loads conditions.**

**FIGURE 5:**
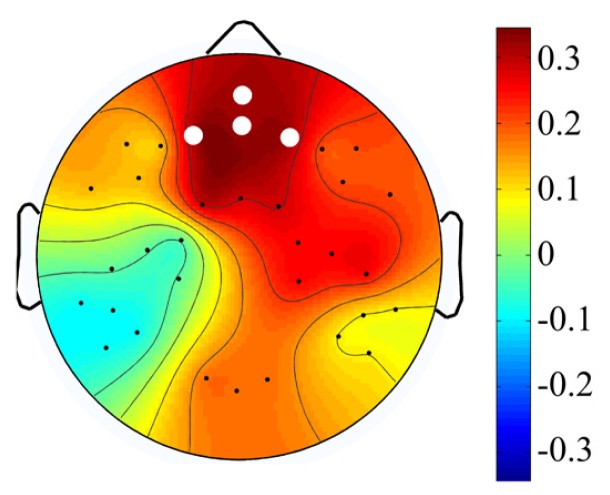
**Scalp distribution of theta – WMC association Coefficients for the correlation between change in theta power and WMC.** White dots indicate cluster with *p* ≤ 0.01.

In order to further examine the relationship between the magnitude of change in theta power and WMC, we computed the difference between mean of theta power in low load conditions (2 and 3) and mean of theta power in high load conditions (4 and 5; for each participant). We found a positive correlation between theta power change from low to high conditions and WMC measured by OSPAN (*r* = 0.32, *p* = 0.008, **Figure 4**). To check the spatial extend of this effect we examined the same correlation between OSPAN and change in theta power in the eight other clusters of electrodes (see Apparatus and Material and **Figure 1**). Scalp distribution of correlation coefficients and their values are presented in **Figure 5** and **Table 3**.

**Table 3:** Correlation coefficients between OSAPN score and theta power increase from lower (2 and 3 digits) to higher (4 and 5 digits) loads for all EEG clusters.

	Frontal			Central			Posterior		
	Left	Middle	Right	Left	Middle	Right	Left	Middle	Right
**OSPAN score**	0.128	0.318**	**0.205**	-0.028	0.285*	0.253*	-0.085	0.179	0.092

**p < 0.001, *p < 0.05, coefficient in bold p < 0.09.

All theta clusters correlating significantly with OSPAN score (see **Table 3**) were put into regression with backward elimination as an input method. The final model included only one predictor – middle frontal theta power. The R-square for this model was 0.101 [adjusted R-square.088, *F*(1,67) = 7.55, *p* < 0.01] indicating that nearly 10% of variance in OSPAN score could be explained solely by changes in middle frontal theta power. All models with standardized coefficients are described in details in **Table 4**.

**Table 4:** Standardized coefficients and R-squares for subsequent models in backward elimination regression with OSPAN as a dependent variable.

Model	Predictors	Coefficients	R-squares (adjusted)
1	Middle frontal	0.247	0.115 (0.06)
	Right frontal	-0.043	
	Middle central	0.053	
	Right central	0.120	
2	Middle frontal	**0.270**	0.114 (0.073)
	Right frontal	-0.034	
	Right central	0.139	
3	Middle frontal	**0.256**	0.113 (0.087)
	Right central	0.127	
4	Middle frontal	0.318**	0.101 (0.088)

**p < 0.001, coefficients in bold p < 0.09.

## DISCUSSION

The aim of this study was to examine the differences in FM theta response to memory load between low and high WMC individuals. Our results clearly show that WMC is an important factor that explains inter-subject variability in FM theta response to memory demands. In contrast to previously introduced factors such as personality traits or level of anxiety (Inanaga, 1998), WMC is a strictly cognitive individual characteristic. These results may broaden our understanding of the importance of individual differences in WMC and neural mechanisms underlying WM maintenance.

### WORKING MEMORY CAPACITY, MEMORY LOAD, AND THETA OSCILLATIONS

We observed a linear increase of FM theta (4–6 Hz) power in response to memory load, which is in agreement with previous findings regarding neural correlates of WM (Gevins et al., 1997; Jensen and Tesche, 2002; Onton et al., 2005). Interestingly, results from this study showed that load-related psychophysiological processes vary among low and high WMC individuals. Specifically, we found a positive relationship between the increase of theta power from lower to higher memory loads and participant’s WMC (OSPAN score). Moreover, this relationship was restricted to FM region. After splitting all of the participants into two groups with regard to their WMC the linear increase of theta power with memory load were visible only in the high WMC group. This was true for both absolute and relative theta power. Importantly, we found no evidence for differences between high and low WMC groups on behavioral level. This may suggest that stronger initial absolute theta power in low WMC individuals (and therefore no changes across loads – in both absolute and relative theta power) is needed for them to achieve similar to high WMC individuals’ level of task performance. In the following sections we will propose a possible interpretation of our results including already established theories explaining FM theta increase.

### NEURAL EFFICIENCY HYPOTHESIS

As OSPAN is an indicator of cognitive or attention control efficacy (see e.g., Unsworth and Engle, 2005) we could speculate that the neural activity of people with higher OSPAN scores, i.e., with greater cognitive control is regulated more efficiently. Especially that researchers point to the fact that low WMC individuals generally are unable to effectively control various aspects of behavior, such as inhibition of irrelevant stimuli (Vogel et al., 2005) as well as updating memory content (Jiang and Kumar, 2004; Woodman and Vogel, 2005). The updating process interferes with already stored information and therefore both encoding and maintenance is less efficient. We could thus interpret our results in the following way: low WMC individual’s neural activity is less efficient therefore their system is already in an as intense mode of maintenance in load two as when maintaining more information. The disability to successfully clear memory content after it’s no longer needed may contribute to sustaining this mode. High WMC individuals, on the other hand, take advantage of having more efficient neural networks. The neural activity is different between loads because it adjusts to task requirements. We see that the system gradually becomes to work in the intense mode of maintenance (lower theta power for smaller loads which increases linearly with load). Moreover, this could also explain why the differences between max load (load five) and max load – 1 (load four) is less distinct than differences between load four or five and other loads – at some point high WMC people achieve a “full swing” of their FM-theta related neural network activity.

#### Management and efficiency: allocation of resources

Frontal theta increase is perceived as a manifestation of cognitive demands required for successfully completing a complex task. These demands have been linked to focused attention and allocation of resources (Missonnier et al., 2006; Sauseng et al., 2007; Mitchell et al., 2008). Dehaene et al. (1998) introduced an interesting concept of how recruitment of necessary resources takes place. In general, the dynamically developing network approach puts a lot of accent on perceiving brain activity as cooperation between elements (nodes) forming networks responsible for specific processes rather than activity of unrelated and independent structures (Bressler and Menon, 2010). Dehaene et al. (1998) proposed a global workspace – a widespread network of neurons in different brain regions that is activated when task is demanding and the “indigenous” processing units need more support. Furthermore, they put forward that communication between nodes of this global workspace is via theta oscillations. Increase of frontal theta power registered from the surface might be a manifestation of global workspace being activated. Hence, low WMC individuals probably have to engage more resources in the low loads – in contrast to high WMC individuals they activate the global workspace even when the task is easy.

### MENTAL EFFORT

Mental effort has been also indicated as a possible explanation of the enhancement of FM theta signal (Gevins et al., 1997; Onton et al., 2005). This approach is in our opinion strongly connected to neural efficiency. According to this perspective theta should be seen not so much as an indicator of the amount of information stored in memory but as an index of effort one puts into remembering them. Of course, in most cases there is a positive relation between memory load or number of elements kept in memory and the effort one has to put in the task, but this assumption is especially important when we consider individual differences in the ability to control attention. This suggests that for low WMC individuals Sternberg task requires more mental effort even in the easiest condition, whereas high WM individuals’ mental effort increases with task difficulty (load). The reason why this task becomes more effortful is because the efficiency of the system that works on its completion is lower. Therefore, we think that our neural efficiency hypothesis is in line with the mental effort theory, and provides an interesting addition to it.

## CONCLUSION

This study shows that oscillatory activity may differentiate people characterized by high and low WMC. The distinct patterns of high and low WMC individual’s theta response to memory load, and more importantly, theta power increasing from lower to higher memory loads as a function of WMC, seem to support the assumption that theta activity during maintenance reflects not only the amount of information stored, but also the effort it takes to remember them and the efficiency of involved neural processes. This is probably due to system’s ability to update information and distribute resources needed to store them. Individual alternations of theta response to memory load are dependent on individual differences such as WMC. This contributes to perceiving FM theta as an individual trait, which can reflect neural efficiency or individual memory span flexibility.

## Conflict of Interest Statement

The authors declare that the research was conducted in the absence of any commercial or financial relationships that could be construed as a potential conflict of interest.
